# Computational Analysis of Sandwich Panels with Graded Foam Cores Subjected to Combined Blast and Fragment Impact Loading

**DOI:** 10.3390/ma16124371

**Published:** 2023-06-14

**Authors:** Lang Li, Fan Zhang, Jiahui Li, Fusen Jia, Bin Han

**Affiliations:** 1 School of Mechanics Civil Engineering and Architecture, Northwestern Polytechnical University, Xi’an 710072, China; lilang@nwpu.edu.cn (L.L.);; 2 School of Mechanical Engineering, Xi’an Jiaotong University, Xi’an 710049, China

**Keywords:** sandwich panel, graded foam, combined loading, optimal gradient

## Abstract

This study aimed to evaluate the performance of sandwich panels with graded foam cores of layered densities against combined blast and fragment impact loading, and to ascertain the optimal gradient of core configuration that would maximize the performance of sandwich panels against combined loading. First, based on a recently developed composite projectile, impact tests of the sandwich panels against simulated combined loading were conducted to provide a benchmark for the computational model. Second, a computational model, based on three-dimensional finite element simulation, was constructed and verified by means of a comparison of the numerically calculated and experimentally measured peak deflections of the back facesheet and the residual velocity of the penetrated fragment. Third, the structural response and energy absorption characteristics were examined, based on numerical simulations. Finally, the optimal gradient of core configuration was explored and numerically examined. The results indicated that the sandwich panel responded in a combined manner involving global deflection, local perforation and perforation hole enlargement. As the impact velocity increased, both the peak deflection of the back facesheet and the residual velocity of the penetrated fragment increased. The front facesheet was found to be the most important sandwich component in consuming the kinetic energy of the combined loading. Thus, the compaction of the foam core would be facilitated by placing the low-density foam at the front side. This would further provide a larger deflecting space for the front facesheet, thus reducing the deflection of the back facesheet. The gradient of core configuration was found to have limited influence on the anti-perforation ability of the sandwich panel. Parametric study indicated that the optimal gradient of foam core configuration was not sensitive to time delay between blast loading and fragment impact loading, but was sensitive to the asymmetrical facesheet of the sandwich panel.

## 1. Introduction

Blasts occur in many situations, such as on battle fields, in terrorist attacks and in industrial accidents, greatly threatenings military equipment and buildings [1,2,3,4,5]. Constructing protective structures and devising strategies to withstand blast impacts have been hot topics for many years. In the past two decades, sandwich structures, such as the foam core sandwich panel, have gradually been utilized as protective structures, due to their outstanding performances in resisting blast and projectile impact loading [6,7,8]. With growing requirements for enhanced blast resistance and perforation resistance from foam core sandwich panels, a variety of strategies have been proposed, such as asymmetrical facesheet design, core configuration design, etc. [9,10]. Introducing density gradient to the foam core has been demonstrated to be an effective strategy in the resistance of sandwich panels against individual fragment impact loading and blast loading [11]. However, in realistic blasts, protective structures must not only withstand individual loading but also withstand encounters with combined blast and fragments impact loading [12,13]. It is not yet known whether density-graded foam core exhibits outstanding performance under combined loading. Therefore, it is necessary to explore the performance of graded sandwich panels under combined loading.

Under blast loading conditions, the density gradient of the foam core has a considerable effect on the crushing mode of the core, which further changes the deformation modes of sandwich panels, and, thus, changes their anti-blast abilities. Recently, the blast resistance of sandwich panels and sandwich shells with density-graded foam cores have been investigated. Liu et al. [14] and Jing et al. [15] numerically explored the blast resistance of an all-metallic layered graded sandwich panel against air blast loading of a certain impulse. Xiang et al. [16,17], Sun et al. [18], Jing et al. [19] and Zhou et al. [20] examined the blast resistance of all-metallic layered graded sandwich panels in a wide range of impulses. Zhou et al. [21], Yang et al. [22] and Zhou et al. [23] explored the blast resistance of sandwich panels with graded PVC foam cores. It was shown that the blast resistance of a graded sandwich panel was sensitive to core gradient types (layered or continuous), the bonding strength between each layer, core material types, thicknesses of facesheets and blast intensities. The blast resistance of sandwich shells has also been widely explored. Jing et al. [24] explored the anti-blast ability of graded cored cylindrical sandwich shells under outside air blast loading. Li et al. [25] explored the blast resistance of spherical sandwich shells with graded foam cores subjected to inner blast loading. Liang et al. [26,27] explored the anti-blast abilities of graded cored sandwich rings against inner blast loading. Their studies indicated that the proper arrangement of the core gradient would enhance the anti-blast abilities of sandwich shells. Recently, the optimal designs of graded sandwich panels [28,29] and sandwich cylindrical shells [30] were investigated, with the results indicating that graded sandwich panels/shells have advantages in blast resistance over their ungraded counterparts under air blast loading.

In addition to the anti-blast abilities of graded sandwich structures being widely investigated, the anti-perforation abilities of graded sandwich structures has also attracted a lot of attention. Feng et al. [31] explored the effect of core density on the damage resistance of sandwich panels, and showed that the influence was dependent on the flexural stiffness of the facesheet. Zhou et al. [32] investigated the impact response of sandwich panels with graded PVC foam, finding that placing the foam core with high density at the impact side led to improved perforation resistance. Kazemi et al. [33], Mohammad et al. [34,35] and Nia et al. [36] explored the high velocity impact resistance of sandwich panels with density-graded polyurethane foam cores of three and four layers. It was shown that the ballistic limit of the graded sandwich panel was higher than that of its ungraded counterpart, and placing the low-density foam layer at the impact side led to better perforation resistance. Jing et al. [37,38] investigated the low velocity impact resistance of a sandwich panel with a density-graded aluminum foam core. The results indicated that the perforation resistance of the graded sandwich panel was weaker than that of the ungraded sandwich panel, and the arrangement of the density gradient did not obviously improve the perforation resistance.

The damage mechanism of combined loading is quite different from individual blast loading or projectile impact loading. It was reported that combined loading induced synergistic damage on a target [39,40,41,42], which was much more serious than the sum of damages induced by individual loadings [43,44]. Several works explored the performance of sandwich panels under combined loading [45,46,47,48]. The results indicated that the structural responses and energy absorption capacities of sandwich panels under combined loading were quite different to those under individual loading. However, the core configurations in these studies were all uniform. Sandwich panels with graded cores under combined loading have not yet been considered. Therefore, whether the beneficial aspects of graded cores against individual loading still exist under combined loading requires elucidation.

In previous studies, both uniform and graded core sandwich panels under individual loading and uniform core sandwich panels under combined loading have been considered. This study aimed to investigate the performance of graded sandwich panels against combined loading and to ascertain the optimal gradient arrangement of the foam core configuration to maximize the resistance of sandwich panels against combined loading. The investigation was based on a validated computational model. In Section 2, the impact test conducted to provide a benchmark for the computational model is described. In Section 3, the computational model is presented and validated. In Section 4, the performance and optimal gradient of the foam core configuration of the sandwich panel are presented and discussed. Finally, in Section 5, the main results obtained are summarized.

## 2. Experimental Procedures

### 2.1. Specimens

**Figure 1:**
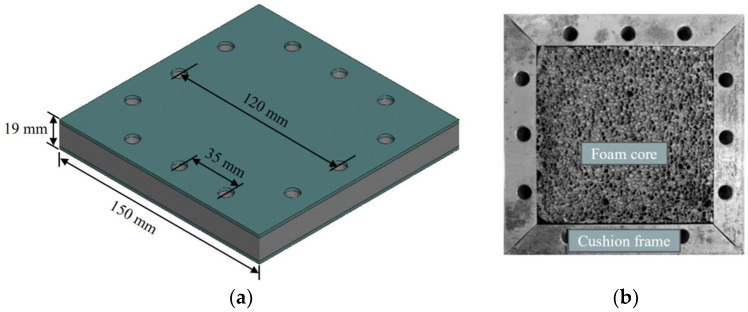
(**a**) Sketch of sandwich specimen; (**b**) Foam core and cushion frame.

**Figure 2:**
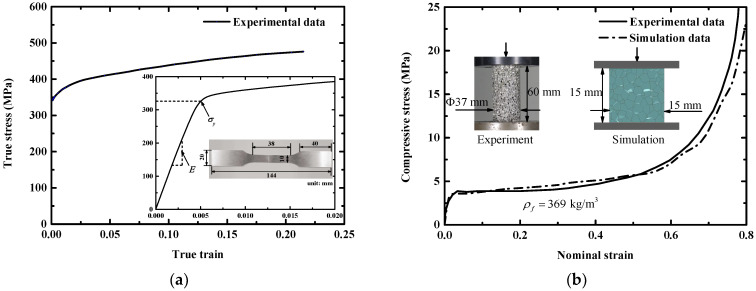
Measured quasi-static stress in terms of the strain responses of component materials: (**a**) uniaxial tensile stress–strain curve for the AA6061-T6 aluminum sheet, (**b**) uniaxial compressive stress–strain curve for the aluminum foam (experimental data was obtained from ref. [42], whereas the simulation data was obtained from compression of the Voronoi foam model constructed in Section 3).

Square sandwich panels made of facesheets, aluminum foam cores, and cushion frames were prepared for the impact tests, as shown in Figure 1a. The facesheets were made of a 6061-T6 aluminum alloy panel (density 2.785 g/cm^3^) with a thickness of 2 mm and an edge length of 150 mm. The tensile stress versus strain curve of the 6061-T6 sheet (2 mm) was measured at a strain rate of 0.001 s^−1^, as shown in Figure 2a, displaying a yield stress of 324 MPa and Young’s modulus of 72 GPa, respectively. The core layer was made of aluminum foam with a thickness of 15 mm and a square edge length of 100 mm. The aluminum foam exhibited an average cell size of 3.2 mm and its average density was 0.369 g/cm^3^. The crushing stress–strain curve was obtained from our previous test [42], in which the plateau stress was 4.5 MPa and the densification strain was 0.7, as displayed in Figure 2b. The foam core was surrounded by steel cushion frames (15 mm in thickness and 25 mm in width), as shown in Figure 1b. The cushion frames were used to provide support to the facesheets and to fasten the facesheets to the base frame. To clarify the damage modes of each component of the sandwich panel, the facesheets and foam core were not bonded by adhesive [49].

### 2.2. Experimental Method

**Figure 3:**
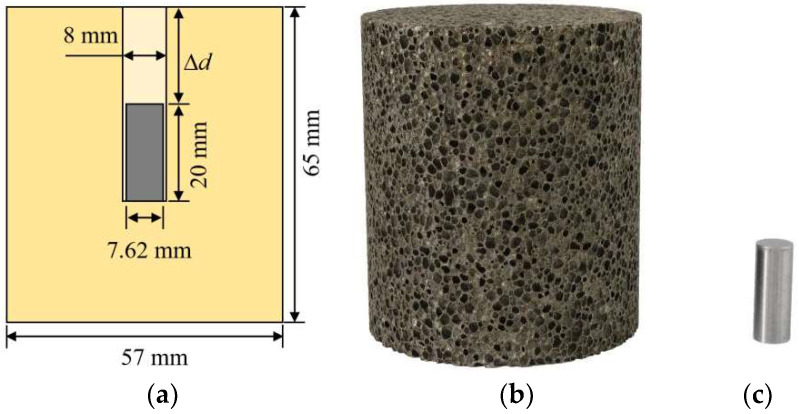
(**a**) Sketch of composite projectile; (**b**) Foam projectile; and (**c**) Blunt-nosed FSP.

A recently developed composite projectile was used to conduct the tests on the sandwich panels subjected to simulated combined loading [42]. The composite projectile was made of an aluminum foam projectile with an embedded FSP (Fragment Simulating Projectile), as shown in Figure 3. The foam projectile and foam core were cut from the same foam block, thus, having the same material properties, whereas the embedded FSP was made of hardened steel. The geometrical sizes of the foam projectile and the FSP are given in Figure 3a. The depth Δd between the foam projectile and the FSP represented the time delay between blast loading and fragment impact loading, and Δd=0 mm was considered in the test.

**Figure 4:**
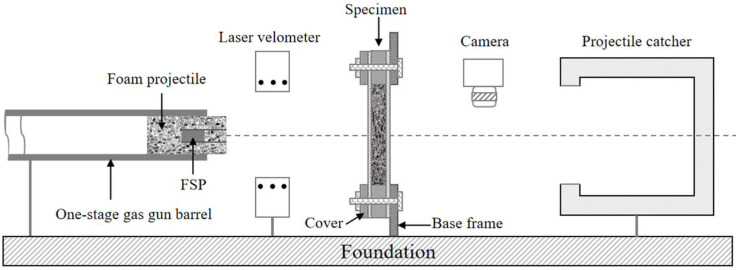
Schematic of the experimental set-up.

The experimental setup is shown in Figure 4. The sandwich specimen was fastened to the base frame with M8 bolts, which left a square area with an edge length of 100 mm exposed in the center. The combined loading was generated via the high-velocity impact of the composite projectile. A single-stage gas gun, with a barrel diameter of 57 mm, was used to accelerate the composite projectile. The impact velocity and residual velocity of the FSP were measured by a laser velometer and a high-speed video camera, respectively.

## 3. Computational Model

### 3.1. Model Description

**Figure 5:**
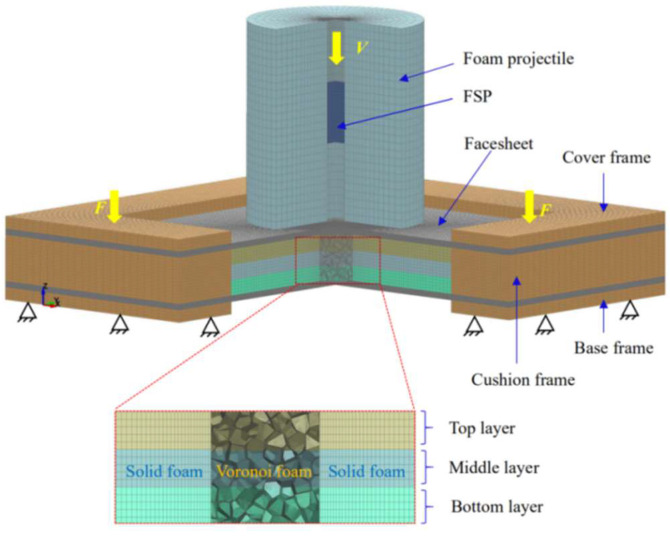
Numerical model (three-quarters of the model is presented) of layered foam core sandwich panel under composite projectile.

The performance of layered graded sandwich panels against combined loading was investigated in LS-DYNA. The finite element model was constructed (as seen in Figure 5), and the geometry of the sandwich panel was the same as that of the specimen. The numerical model consisted of three parts: the composite projectile, the sandwich panel and the fixture. The composite projectile consisted of an aluminum foam projectile and an embedded FSP, and aimed to simulate combined loading on the sandwich panel. The sandwich panel consisted of facesheets and a layered density graded foam core, in which the foam core was divided into three layers. Since the failure strength of the adhesive epoxy employed between two adjacent foam layers in applications was generally as strong as the fracture strength of the aluminum foam, it was assumed that each two adjacent foam layers was perfectly bonded (in the present study, common node constraints were utilized). The fixture consisted of a base frame, cushion frame and cover frame, and aimed to simulate the fixed boundary.

**Figure 6:**
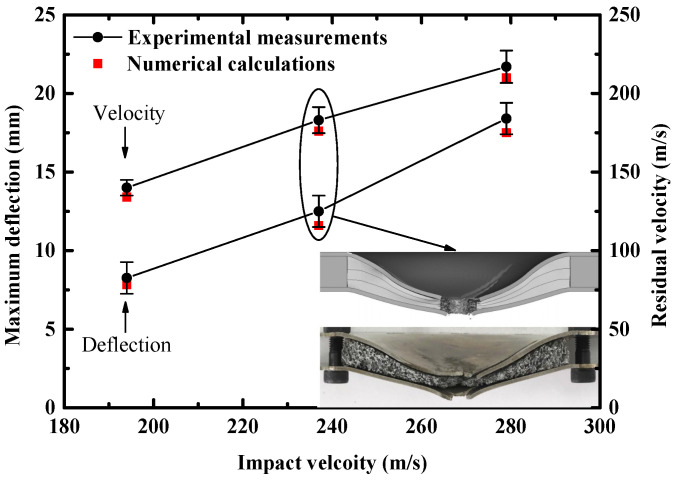
Experimentally measured and numerically calculated residual velocity of FSP and peak deflection of the back facesheet of the sandwich panel under different impact velocities. Note: the cross-sectional views of the deformation profiles of the sandwich panel after impact (237 m/s) are illustrated.

The composite projectile, the facesheets and the frames were all modeled by means of eight-node brick element with reduced integration. According to the experimental observed damage mode of the sandwich panel, shown in Figure 6, the central foam core underwent both penetration and compression, while the surrounding foam core underwent only compression. Thus, the foam core was modeled in a multi-scale manner. The central square part, with a side length of 15 mm, was modeled by 3D-Voronoi cellular shells (i.e., Voronoi foam as given in Appendix A), whereas the rest of the core was modeled by a solid entity. A mesh convergence study was performed to determine the mesh size of each component of the numerical model. The numbers of elements in the longitudinal and circumferential directions of the foam projectile were 15 and 32, respectively. Two mesh sizes were used in the radial direction, with the element size being 0.5 mm in the region from 0 to 4 mm. A varying mesh size with 12 elements was used from 4 mm to 28.5 mm. A global mesh size of 0.5 mm was employed for the FSP. The facesheets were divided into two regions. The mesh size in the central square region, with a side length 15 mm, was 0.3 mm in length, while in the square ring region outside of the central square region a varying mesh size was employed (from inside 0.3 mm to outside 2 mm), and through the thickness of the facesheet there were 10 elements. Global mesh sizes of 1 mm and 2 mm were employed for the frames through the thickness direction and in-plane, respectively. The Voronoi foam core was meshed by means of four-node shell elements (an element size of 0.2 mm was employed) with 5 integration points through thickness, whereas the solid foam core was meshed by eight-node brick elements (an average element size of 1 mm through thickness direction and 0.5 mm in plane was employed) with reduced integration.

No bonding was employed between the facesheet and the core, which was consistent with the specimen. A tied node to surface contact was employed between the boundary of the Voronoi foam core and the inner boundary of the surrounding solid foam core. An automatic surface to surface contact was set for all possible surface to surface contacts (the friction value of 0.2 was employed) including: (i) contact between each of the two foam core layers, (ii) contact between the core and the surrounding cushion frame, (iii) contact between the cushion frame and facesheet, (iv) contact between the facesheet and base/cover frame, and (v) contact between the facesheet and foam core. An automatic single contact was employed to avoid self-penetration of the cell walls of the Voronoi foam core. The base frame was fixed by means of constraint at each bolt hole region, whereas a force was exerted on each bolt hole region in the cover frame to sandwich the specimen. In the simulation, the composite projectile impacted the sandwich panel with an initial velocity. Between the sandwich panel and composite projectile, an automatic node-to-surface contact was employed. It should be noted that the failure between the facesheet and foam core could not be considered, based on the present model.

### 3.2. Constitutive Model

The facesheets of the sandwich panels were made of aluminum alloy (AA6061-T6), the dynamic behavior of which was simulated by the Johnson–Cook model [50]. The equivalent stress was expressed as follows:

(1) $$\sigma_{eq}=\left(A+B\varepsilon_{e}^{n}\right){\left(1+{\dot{\varepsilon }}_{e}^{*}\right)}^{c}\left(1-T^{*}{}^{m}\right)$$

where ${\dot{\varepsilon }}_{e}^{*}={\dot{\varepsilon }}_{e}/{\dot{\varepsilon }}_{0}$ was the normalized equivalent plastic strain rate, in which ${\dot{\varepsilon }}_{0}$ was a reference strain rate, and c was the strain rate coefficient; A was the yield stress (quasi-static), n and B were strain hardening coefficients; the homologous temperature and thermal softening coefficient was donated by $T^{*}=\left(T-T_{r}\right)/\left(T_{m}-T_{r}\right)$ and m, respectively, where T, $T_{m}$ and $T_{r}$ was, in turn, the absolute temperature, the melting temperature and the reference temperature.

Table 1 lists the J-C parameters of AA6061-T6 that were taken from Ref. [51], whereas the failure criterion with maximum shear strain values of 0.5 was adopted for AA6061-T6.

The foam core was made of aluminum foam, of which the aluminum cell wall material of Voronoi foam core was represented using a bilinear strain-hardening model:

(2) $$\sigma =\left\{\begin{array}{ll} E_{s}^{f}\varepsilon , & \varepsilon \leq \frac{\sigma_{ys}^{f}}{E_{s}^{f}}\\ \sigma_{ys}^{f}+E_{t}^{f}\left(\varepsilon -\frac{\sigma_{ys}^{f}}{E_{s}^{f}}\right), & \varepsilon >\frac{\sigma_{ys}^{f}}{E_{s}^{f}} \end{array}\right.$$

where $E_{s}^{f}$ was the Young’s modulus and $E_{t}^{f}$ was the tangent modulus, respectively, and $\sigma_{ys}^{f}$ denoted the yield stress. Detailed mechanical properties are listed in Table 2. The aluminum cell wall material was regarded as having failed when the maximum shear strain reached 0.3. The solid foam core was modeled by the crushable foam material model. The crushing stress–strain curve for each foam layer of different density was obtained by multiplying the measured stress–strain curve in Figure 2b with a coefficient, thereby making the plateau stress of each foam layer match the following relation [52]:

(3) $$\sigma_{\rho_{i}}/\sigma_{\rho_{j}}=0.35{\left(\rho_{i}/\rho_{j}\right)}^{0.15}$$

where $\sigma_{\rho_{i}}$ and $\sigma_{\rho_{j}}$ were the plateau stress of foam layer with density $\rho_{i}$ and $\rho_{j}$, respectively. The input elastic modulus of each foam layer was obtained from the elastic regime of the corresponding crushing stress–strain curve. The Poisson’s ratio of aluminum foam was generally ignorable [28,29,30], thus a zero value was assumed in the present study.

The FSP was made of hardened steel and the frames were made of die steel. As the strength of the hardened steel and die steel were relatively high compared to that of the aluminum alloy and aluminum foam, it was assumed that both the FSP and the frames were rigid, and, thus, a rigid material model was employed for the FSP and the frames. The density and Young’s modulus were 7.8 g/cm^3^ and 200 GPa, respectively. The foam projectile was also modeled by the crushable foam material model, in which the input crushing stress–strain curve was taken from the measured stress-strain curve shown in Figure 2b, and the Poisson’s ratio was set to zero.

## 4. Results and Discussions

### 4.1. Validation of Computational Model

To provide a benchmark to the numerical approach, three sandwich panels with uniform density cores (0.369 g/cm^3^) were subjected to composite projectiles (Δd=0 mm) of different impact velocities. The measured incident velocities of the three sandwich panels were 193 m/s, 237 m/s and 279 m/s, respectively. The peak deflection of the back facesheet and the residual velocity of the FSP are two key criteria in the anti-blast design and the anti-perforation design of sandwich panels [14,32]. Therefore, these two criteria were selected to validate the effectiveness of the numerical model. The residual velocities of the FSP and maximum central deflections of the back facesheet obtained from experimental measurements and numerical calculations are compared in Figure 6. The errors of the experimental measured residual velocities were ±3.5%, ±4.6%, and ±5.3%, respectively, due to oblique shooting by the high-speed camera. Whereas the errors of the measured maximum central deflections of the back facesheet were ±1 mm, i.e., half the thickness of the back facesheet. It was shown that the simulations agreed well with the measurements for both residual velocity and deflection. Therefore, the above numerical model was considered effective to simulate perforation and deflection behaviors of the sandwich panel when subjected to combined loading.

### 4.2. Structural Response

**Figure 7:**
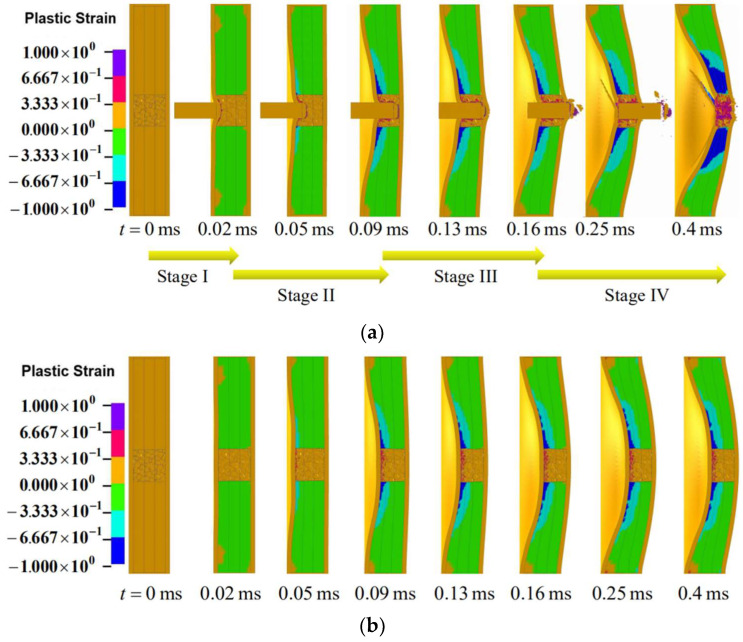
Cross-sectional view of numerically simulated foam-cored sandwich panel under (**a**) combined loading (Foam projectile was not shown here. Only the FSP was displayed) and (**b**) only blast loading in time sequence.

As has been reported in the introduction, combined loading induces synergistic damage to structures [39,40,41,42]. In order to reveal the synergistic damage features of sandwich panels under combined loading, the cross-sectional view of the layered graded sandwich panel (the density of each core layer was 0.369 g/cm^3^) under both combined loading and blast loading at various instants are displayed in Figure 7. The two loadings employed the same foam projectile. It was noticed that the sandwich panel responded in a global deflection manner under blast loading, while in a combined manner involving global deflection, central perforation and perforation hole enlargement under combined loading. Moreover, it was also shown that the deflection of both facesheets under combined loading at each time instant was larger than that under blast loading, indicating that the combined loading induced enhanced damage to the sandwich panel in terms of maximum deflection of the central back facesheet. It was inferred that the enhanced damage was triggered by the perforation hole. After the facesheets were perforated, the perforation hole was further enlarged by the foam projectile in this process. The perforation hole triggered crack propagation in the facesheets and lowered the load-carrying capacity of the facesheets, thereby enhancing the damage to sandwich panel. It was shown that the final densification extent (t=0.4 ms) of the foam core was much larger under combined loading than under blast loading. This was due to the fact that the cracks that occurred were propagated in the front facesheet under combined loading. As the cracks became larger, the foam core was further compacted, thus resulting in larger densification extent.

**Figure 8:**
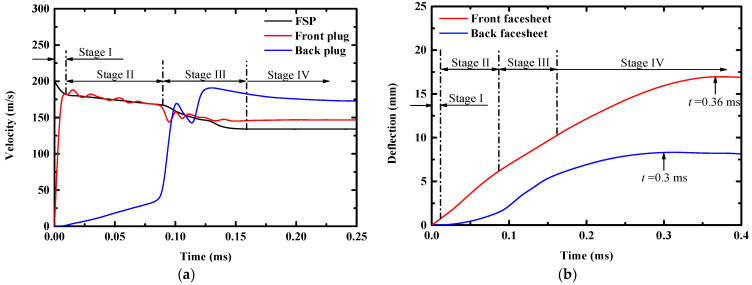
(**a**) Velocity-time histories of the FSP and plugs ejected from the front facesheet (front plug) and the back facesheet (back plug) of sandwich panel; (**b**) Deflection–time histories of the central front and back facesheets of the sandwich panel.

To illustrate the structural response of the sandwich panel under combined loading, the deflection-time histories of the facesheets and velocity–time histories of the FSP are presented in Figure 8a,b, respectively. According to Figure 8a, the perforation process of the FSP could be divided into four stages. In stage I, the front facesheet was perforated in plugging mode, a plug (front plug) was ejected and attained the same velocity as the FSP. In stage II, the foam core was penetrated by the FSP and the plug ahead. In stage III, the back facesheet was extruded by the densified foam core ahead of the ejected plug and FSP until penetrating the back facesheet, and a plug (back plug) was also ejected from the back facesheet. In stage IV, the FSP left the sandwich panel. The deflection of the front facesheet induced by the foam projectile (blast loading) lasted during the entire response process, as seen in Figure 8b. The front facesheet started to deflect as the foam projectile interacted with it (t=0 ms), meanwhile the front facesheet attained velocity and started to compact the foam core. An elastic precursor wave was first formed in the foam core and propagated to the back facesheet, and, thus, the back facesheet deflected gradually (stage II). With continued compaction of the foam core, a shock wave formed in the foam core and propagated to the back facesheet. The back facesheet was then clearly deflected (stage III). After the deflection of the back facesheet achieved a critical value, the deflection rate of the back facesheet slowed until reaching the maximum deflection. The back facesheet reached its maximum deflection at t=0.3 ms, while the front facesheet reached its maximum deflection at t=0.36 ms. During this regime (0.3 ms~0.36 ms), the foam core was further compacted to a larger densification strain by the front facesheet, as analyzed above.

There was a coupling effect between global deflection and local perforation. For example, the foam projectile was actually hitting a sandwich panel with a perforation hole, and the perforation hole affected the deflection by lowering the load carrying capacity of the sandwich panel. The FSP was actually penetrating a deflected sandwich panel, and the deflection delayed the perforation process, changing the perforation resistance of the sandwich panel, a detailed explanation of which can be found in Ref. [52].

### 4.3. Energy Absorption Mechanism

**Figure 9:**
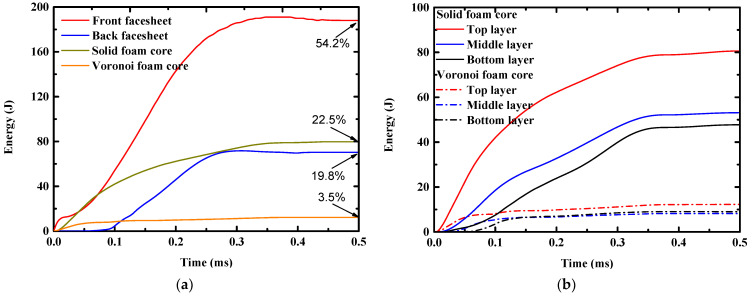
Internal energy time histories of (**a**) each sandwich component; and (**b**) each layer of foam core.

To further reveal the inherent mechanism of the deflection and perforation responses of graded sandwich panels, the internal energy time histories of each component of the sandwich panel were obtained numerically and compared, as shown in Figure 9a. Generally, the internal energy could be partitioned into three components: elastic strain energy, plastic dissipated energy, and damage energy owing to fracture. The elastic strain energy could be considered the “recoverable” energy, while the final constant value in the curves represented the energy absorbed by each component in the impact process [28]. It was shown that the front facesheet absorbed approximately more than half (54.2%) of the total energy absorbed by the sandwich panel, followed by the foam core (26%), and finally the back facesheet (19.8%).

Figure 9b compares the energy absorbed by each layer of the foam core. The top foam layer absorbed more energy than the other foam layers, whereas the bottom foam layer absorbed the least energy, although the thickness and density of each foam layer was the same. According to the cross-sectional profile of the sandwich panel in Figure 7, the top foam layer was compacted to a larger extent than the other foam layer, and, therefore, the top layer absorbed more energy. It was also shown that the solid foam core absorbed more energy than the Voronoi foam core. Considering that the solid core underwent only compression, whereas the Voronoi core underwent combined compression and perforation, it was inferred that the foam core absorbed most energy in a compression manner.

### 4.4. Parametric Study

In this section, the parametric study is described. The conducted parametric study was based on the validated numerical model. The influence of gradient type of the foam core on the performance of the sandwich panel against combined loading was examined, and the sensitivity of the optimal core gradient to the time delay and asymmetrical facesheet explored.

#### 4.4.1. Effect of Gradient Type for Foam Core

In order to explore the influence of the foam core gradient on the performance of the sandwich panel, the density gradient, through thickness of the sandwich panel, was considered. Three core densities were considered: 0.519 g/cm^3^ (*H*), 0.369 g/cm^3^ (*M*) and 0.219 g/cm^3^ (*L*). Therefore, there were six different foam core configurations. To facilitate comparison, the average density of the foam core was kept constant (i.e., 0.369 g/cm^3^). The graded core layer arrangements of the sandwich panel were named by a combination of *H, M* and *L,* so, for example, *LHM* indicated that the graded core layer was 0.219 g/cm^3^ (*L*), 0.519 g/cm^3^ (*H*) and 0.369 g/cm^3^ (*M*) from top (front faceshsst) to bottom (back facesheet).

**Figure 10:**
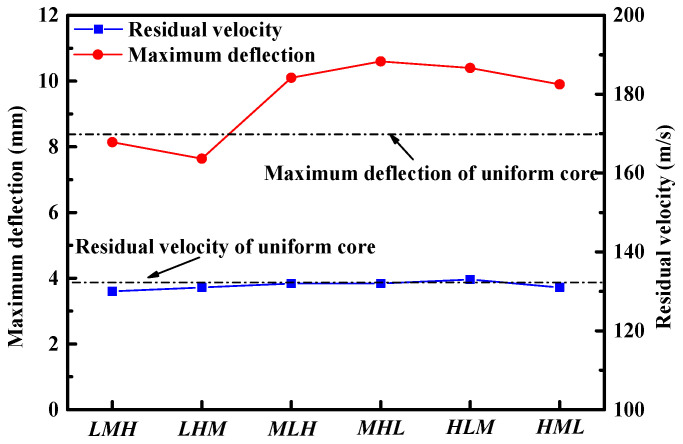
The maximum back facesheet deflection of sandwich panels and residual velocity of the FSP for different core configurations.

Here, we discuss sandwich panels with graded foam cores subjected to combined loading (Δd=0 mm) of impact velocity of 200 m/s. The residual velocity of the FSP and the peak deflection of the central back facesheet for different core configurations are plotted in Figure 10. It was shown that the sandwich panel with the *LHM* core exhibited smaller peak deflection of the back facesheet than was evident with the other core configurations. For example, the peak deflection for *LHM* was 8.7% smaller than the uniform core configuration, indicating that a well-designed density-graded foam core outperformed its uniform counterparts in terms of blast resistance. Unlike the maximum deflection of the back facesheet, the FSP’s residual velocity was insensitive to the core configurations. The graded foam cored sandwich panel exhibited nearly the same perforation resistance as the uniform cored sandwich panel.

**Figure 11:**
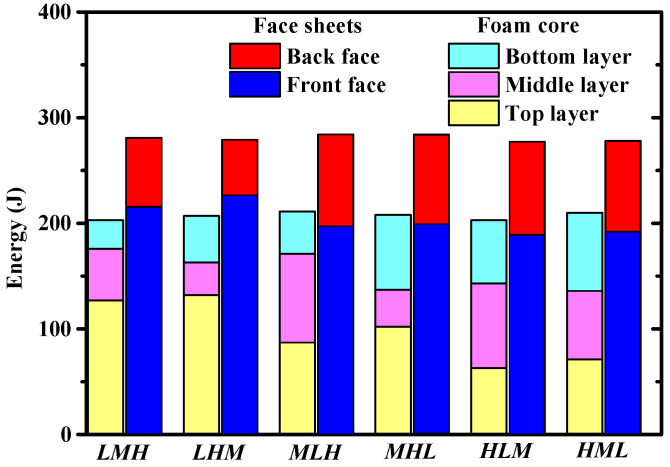
Energy absorbed by each component of sandwich panels with different graded foam cores.

**Figure 12:**
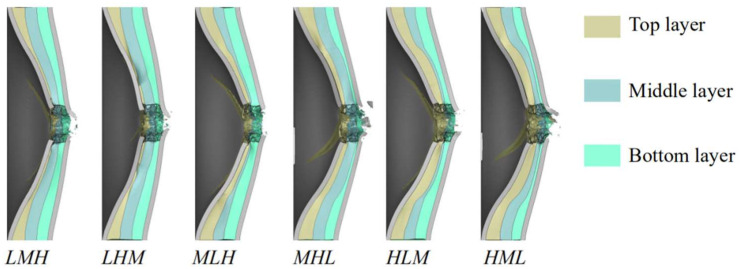
The cross-sectional view of final deformation profiles of sandwich panels with different graded foam cores.

The energy absorbed by each component of the sandwich panels having different core layer arrangements can be observed in Figure 11. It was shown that the total energy absorbed by the facesheets was nearly the same, but the ratios of energy absorbed by the front facesheet and the back facesheet were different for different foam core configurations. The energy absorbed by the back facesheet of *LHM* was the smallest among the considered core configurations, which was consistent with the trend of the maximum deflection of the back facesheet, shown in Figure 10. Placing low density foam (*L*) at the top core layer led to more energy being absorbed by the top core layer, whereas placing the high-density foam (*H*) at the top layer led to less energy being absorbed by the top foam layer. This feature was also applicable for the bottom layer and the middle layer. Therefore, placing low density foam at the top layer was beneficial in enhancing the blast resistance of the sandwich panel. Actually, placing low density foam at the top layer would facilitate the compaction of the foam core, as seen in Figure 12, which further facilitated the deflection of the front facesheet, as more kinetic energy of the combined loading was absorbed by the front facesheet, so the deflection of the back facesheet reduced. Comparison between *LMH* and *LHM* indicated that placing high-density foam at the middle layer provided stronger support to the top layer, and the top layer would be compacted to a larger densification strain. As a result, the front facesheet of *LHM* had a larger deflection and the back facesheet of *LHM* had a smaller deflection.

#### 4.4.2. Effect of Time Delay

**Figure 13:**
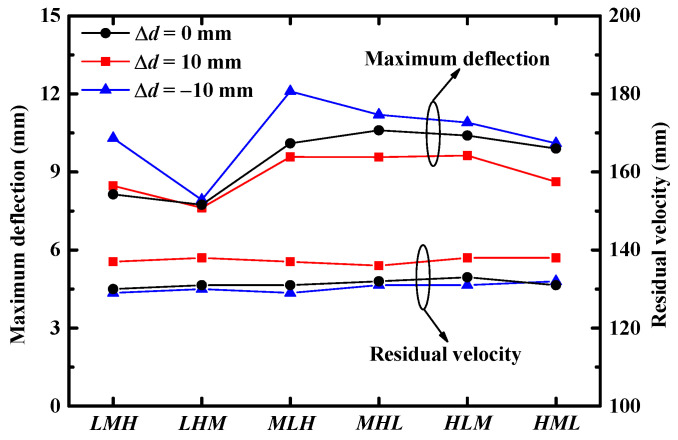
The maximum deflection of back facesheets of sandwich panels and residual velocities of the FSPs for different core configurations subjected to combined loading with different time delays between blast and fragment impact loading (Δd).

The above analysis indicated that a sandwich panel with a core configuration of *LHM* exhibited the best blast resistance, i.e., minimum deflection of the back facesheet. However, whether this conclusion was applicable for different combined loadings was not known. It has been reported that time delay between blast loading and fragment impact loading is one of the most important characteristics of combined loading [42,44]. Therefore, sandwich panels with different core configurations and with three different time delays, i.e., Δd=−10 mm (blast loading acts before fragment impact loading), Δd=0 mm and Δd=10 mm (fragment impact loading acts before blast loading), were subjected to combined loading. The peak deflection of the back facesheet and the residual velocity of the FSP for various core configurations under combined loading and with different time delays (Δd) are illustrated in Figure 13.

According to Figure 13, the time delay had an obvious influence on both the residual velocity of the FSP and the maximum deflection of the back facesheet. The sandwich panel exhibited superior blast resistance (smaller maximum deflection of back facesheet) under combined loading of Δd=10 mm than under combined loading of Δd=0 mm and Δd=−10 mm for most core configurations. As for the perforation resistance (residual velocity of the FSP) of the sandwich panel, the sandwich panel exhibited superior perforation resistance (smaller residual velocity of the FSP) under combined loading of Δd=−10 mm. The difference in perforation resistance for each sandwich panel could have been due to a deforming effect [52]. The FSP was penetrating a deforming sandwich panel (induced by blast loading), and, as the deforming extent was determined by the time delay, different time delays would result in different perforation resistances of the sandwich panels. It was shown that the deforming extent of the sandwich panel under combined loading of Δd=−10 mm achieved a superior perforation resistance ability. Although the blast resistance and perforation resistance were sensitive to time delay, the perforation resistance of the sandwich panel was not sensitive to core configuration for all the considered combined loadings. The optimal gradient of core configuration, in terms of blast resistance, was still *LHM*.

#### 4.4.3. Effect of Asymmetrical Facesheets

**Figure 14:**
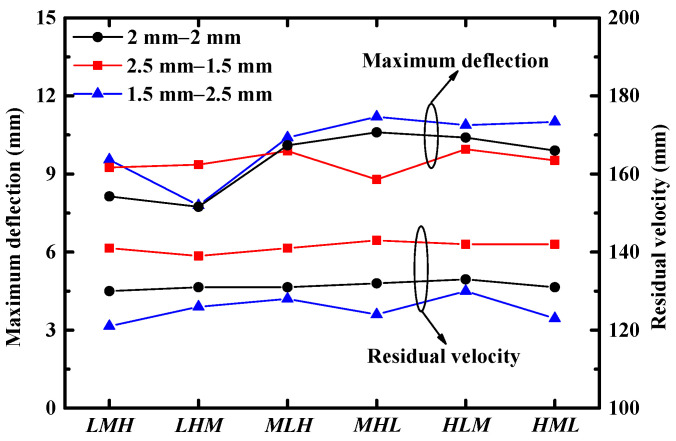
The maximum back facesheet deflection and residual velocity of the FSP for different core configurations and different asymmetrical facesheets subjected to combined loading.

In the above sections, all of the sandwich panels considered had symmetrical facesheets. In practical applications, asymmetrical facesheets are generally applied to achieve better performance [9,11]. Therefore, whether asymmetrical facesheets would affect the optimal gradient of the core configuration was explored. Three pairs of asymmetrical facesheets were considered, in which a total thickness of 4 mm was employed (x−y in Figure 14 denotes that the front and back facesheets were *x* mm and *y* mm, respectively). The performances (both deflection and residual velocity) of sandwich panels with different core configurations and different asymmetrical facesheet pairs are illustrated in Figure 14.

It was shown that both blast resistance and perforation resistance of the sandwich panels were sensitive to the asymmetrical ratio under combined loading. The sandwich panel with a thicker front facesheet exhibited superior blast resistance but weaker perforation resistance. For different asymmetrical facesheet pairs, the perforation resistance of the sandwich panel was not sensitive to core configuration, whereas the optimal gradient of core configuration was different for various asymmetrical facesheets. It was shown that sandwich panels with facesheets 2 mm–2 mm and 1.5 mm–2.5 mm achieved the best blast resistance with a core configuration of *LHM*, while the sandwich panels with facesheets 2.5 mm–1.5 mm achieved the best blast resistance with a core configuration of *MHL*. This indicated that the optimal gradient of core configuration was sensitive to asymmetrical facesheets.

## 5. Conclusions

The performance of sandwich panels with graded foam cores of layered density against combined blast and fragment impact loading was investigated numerically. The combined loading was generated by a composite projectile. Impact tests of sandwich panels subjected to composite projectiles were conducted to provide a benchmark for numerical modeling. Based on the verified numerical model, the energy absorption and structural response of graded sandwich panels against combined loading were explored, and the optimal gradient of the core configuration ascertained. The main conclusions are summarized as follows:

- The graded sandwich panel responded in a combined manner, involving global deflection, local perforation and perforation hole enlargement, under combined loading;
- The deflection of the sandwich panel delayed the perforation process, thus changing the perforation resistance of the sandwich panel. The perforation lowered the load carrying capacity of each part of the sandwich panel, thus changing the blast resistance of the sandwich panel;
- Placing low density foam at the top core layer was beneficial in enhancing the blast resistance of sandwich panels. Sandwich panels exhibited the best performance against combined loading with a core configuration of *LHM*.
- It was found that both the time delay of combined loading and asymmetry of facesheets affected the blast and perforation resistances of sandwich panels. The optimal gradient of core configuration was not sensitive to time delay of the combined loading. The optimal gradients of core configuration were all *LHM* for the different time delays considered in the present study. The optimal core configuration was sensitive to asymmetrical facesheets, and the optimal gradient might change as the asymmetrical facesheets ratio varied.

## Figures and Tables

**Table 1:** Parameters of the material model for AA6061-T6 aluminum [51].

$\rho_{s}$ (g/cm^3^)	*G* (GPa)	*A* (MPa)	*B* (MPa)	*n*	*m*	*c*
2.785	27.1	324	114	0.34	1.34	0.002

**Table 2:** Parameters of the material model for cell wall material of aluminum foam [53].

$E_{s}^{f}$ (GPa)	$E_{t}^{f}$ (MPa)	$\sigma_{ys}^{f}$ (MPa)	$\rho_{s}^{f}$ (g/cm^3^)	υ
69	58	100	2.7	0.3

## Data Availability

The data presented in this study are available on request from the references and the corresponding author. The data are not publicly available due to the privacy of program data.

## Appendix A. Modeling of Voronoi Foam

The Voronoi function was utilized to construct the foam model with closed cell, aiming to simulate the foam core in the central region. The detailed method was in accordance with [53]. A number of Num seeds were first randomly distributed in a space, and then the space was divided into Num small spaces (cells) by Delaunay triangulation, with each cell including one seed. The border of the cells then constituted the cell wall of the Voronoi foam. Given that the thickness of the cell walls is t, the Voronoi foam has a density of $\rho_{f}$, which can be expressed as:

(A1) $$\rho_{f}=\frac{\sum_{i=1}^{num}A_{i}\cdot t}{V/m}\cdot \rho_{s}$$

where $A_{i}$ is the area of *i*-th cell wall, n is the number of cell walls, $\rho_{s}$ is the density of cell wall material. Foams of different densities can be built by varying the cell wall thickness t.
